# Financial access to health care for older people in Cambodia: 10-year trends (2004-14) and determinants of catastrophic health expenses

**DOI:** 10.1186/s12939-016-0383-z

**Published:** 2016-06-17

**Authors:** Bart Jacobs, Richard de Groot, Adélio Fernandes Antunes

**Affiliations:** Social Health Protection Programme, Deutsche Gesellschaft für Internationale Zusammenarbeit (GiZ), c/o NIPH, No.2, Street 289, Khan Toul Kork, P.O. Box 1238, Phnom Penh Cambodia; Independent consultant, Florence, Italy; Independent consultant, Luxembourgville, Luxembourg UK

**Keywords:** Older people, Low-income country, Cambodia, Out-of-pocket expenses, Catastrophic health expenses, Impoverishment, Indebtedness, Non-communicable diseases, Social assistance, Rural

## Abstract

**Background:**

Older people make up an increasing proportion of the population in low- and middle-income countries. This brings a number of challenges, as their health needs are greater than, and different from, those of younger people. In general, these health systems are not geared to address their needs, and traditional support systems tend to erode, potentially causing financial hardship when accessing health care. This paper provides an overview of older Cambodians’ financial access to health care over time, using nationally representative data to enable the formulation of appropriate responses.

**Methods:**

Using data from three nationally representative household surveys from 2004, 2009 and 2014, we assess key indicators of financial access to health care for households with older people (aged 60 years or older), and compare these with households without older members. For 2014 data, the determinants of catastrophic health expenses at the 10 and 40 % threshold were determined for older people. Data was stratified by age and place of residence (urban/rural), and analysed using Stata statistical software. Sample weights were calibrated to reflect accurate population composition at the time of the survey. Monetary values for 2004 and 2009 were transformed into 2014 values using annual inflation rate figures.

**Results:**

Care-seeking when sick among older people increased considerably from 2004 to 2014, irrespective of gender or place of residence. There were positive trends in the incidence of catastrophic and impoverishing healthcare expenses over the studied time periods. This was also the case for indebtedness. Rural households with older people were considerable more likely to suffer financial hardship due to health-related expenses than their urban equivalents. In 2014, older people spent 50 % more per month on health care than younger people. Determinants of catastrophic health expenditures among households with older people were residing in a rural area, and having a household member with an illness, especially a non-communicable disease.

**Conclusion:**

In order to make health care more equitable for older people, efforts should be directed to rural areas. Interventions should include improving management of non-communicable diseases at the primary care level, together with a reconfiguration of social health protection schemes to increase the inclusion of older people.

## Background

Low- and middle-income countries (LMIC), especially in Asia, are witnessing a remarkable aging of their societies, often at unprecedented speed. For example, the doubling of the proportion of the population over 65 years old took 26 years in China, compared with 68 years in the USA [1]. Also in China, the number of older people (those aged 60 or older) are expected to outnumber those 0–14 years old by 2020 [2]. This trend in population aging is ascribed to decreasing fertility rates, coupled with increased life expectancies due to better survival rates during childhood and improved health care and social determinants of health [2].

Population aging brings a number of challenges, as older people tend to suffer from multiple non-communicable diseases (NCD) and disabilities [3, 4]. In Vietnam, for example, a cross-sectional survey of medical examinations among older people found an NCD prevalence rate of 77 %, with 39 % suffering multiple NCDs [5]. Older people in LMICs tend to have limited or no pension or elderly support schemes, especially those without previous formal employment [6]. In addition, LMICs are experiencing rapid urbanisation, with the migration of large numbers of young adults to urban areas, potentially undermining social support structures [7].

Moreover, healthcare systems in LMICs tend to be focused on maternal and child health and control of infectious diseases, and are not suited for dealing with the health conditions plaguing older people [3, 8]. The inability of the public health sector to provide for the needs of older people may force them into the unregulated or poorly regulated private healthcare sector [9], where costs can be considerably higher. Thus, older people have higher needs for health care than other age groups, but have a limited ability to access the required services in the public sector. Not surprisingly, studies in LMICs found that households with older people, especially those with chronic NCDs or disabilities, experience higher rates of catastrophic expenditures for health care than other households [10–12].

In Cambodia, older people aged 60 years or more constituted 6.3 % of the population in 2008, a figure expected to increase to 11 % in 2030 [13]. However, little is known about their financial access to health care and how this affects the wellbeing of their families. Therefore, in this paper we provide an overview of Cambodian older people’s financial access to health care, using nationwide representative data. For this purpose, we describe the situation of older people in 2014 concerning incidence of illness, care-seeking for illness, associated expenditures, affordability of these costs and borrowing practices to pay for health care, and compare these with the overall population. We contextualise this information over time by comparing the situation in 2014 with similar data from 2004 to 2009. Lastly, we assess the determinants of catastrophic healthcare expenses for older people, to enable the formulation of appropriate responses to reduce financial hardship when accessing health care.

## Methods

### Data

Data was derived from the Cambodian Socio-Economic Survey (CSES). The CSES is a nationally representative survey of households, with information on household income, expenditures, indebtedness and consumption implemented annually since 2004. The household survey is complemented by diaries for households to record their own daily transactions. The CSES is conducted annually with about 3600 households, with larger sample sizes of approximately 12,000 households every five-years (in 2004, 2009, 2014).

The CSES data includes a detailed consumption module, including out-of-pocket expenditures on health services and medications. In addition, the healthcare module collects information about the health status of all household members in the 30 days prior to the survey, including questions on the incidence of illness, care-seeking behaviour, and disability status.

### Definition of key variables

At the individual level, we constructed or determined the following health-related indicators:

- Seeking care when ill: a person was defined as seeking care when s/he reported seeking a consultation at any kind of healthcare provider in the 30 days prior to the survey.
- Seeking medical care: defined as seeking care at a public or private medical provider, thereby excluding the non-medical sector. Because up to two providers have been recorded for each care-seeking incidence since 2009, if either of those visits was to a public or private medical provider then it was considered to be medical care.
- Provider type: respondents reported the kind of healthcare provider for the first visit and (if more than one visit) the last visit only. The analysis combined all healthcare providers into eight categories, which are comparable across all years. The categories include: public health centres; public hospitals; private hospitals; private clinics; pharmacies and stores selling drugs; home care; traditional healers; and other providers. In addition to these eight categories, results were also more broadly categorised in public providers, private providers, and non-medical providers.
- Severity of illness: reported illnesses were categorised as mild, severe or chronic, with mild implying that the illness did not stop the individual from performing usual activities, while severe meant the inability to performing such activities for at least one day in the last 14 days. A condition was considered chronic or non-communicable if the individual suffered from this condition for more than one year.
- Disability: a person was considered disabled – defined in CSES as restricted or unable to perform an activity in a manner considered normal – when reporting one or more difficulties out of a list of nine activities (seeing; hearing; speaking; moving; feeling or sensing; psychological or behavioural functioning; learning; fits; and others). We removed seeing difficulties, as the rate was inconsistently high in 2009 (2.2 %, compared to 0.62 and 0.70 % in 2004 and 2014, respectively).
- Out-of-pocket health expenditures: defined as the total expenditures on health-related services, supplies and products in the 30 days prior to the survey. Expenditures were converted to monthly values by multiplying by 30.4.
- Health-related transportation expenditures: defined as the amount spent on transportation for healthcare seeking in the 30 days before the survey. This metric was only included in the CSES from 2009 onwards. These values were similarly converted to monthly values by multiplying by 30.4.

To estimate the financial burden of health care on households with older people, we closely followed the calculation methods proposed by Xu [14]. In particular, we considered the following indicators:

- Consumption: the sum of purchases, as well as the value of the consumption of one’s own produce, and goods received as gifts.
- Subsistence consumption: a food share-based reference line was used to estimate household subsistence expenditures. This reference line was defined as the weighted average of equalised food expenditures for households whose food expenditures as a share of total household expenditures were between the 45th and 55th percentile of the population.
- Capacity to pay (CTP): defined as a household’s non-subsistence spending, which equals the monthly household consumption minus subsistence expenditures. For households with food expenditures lower than their subsistence spending, non-food expenditures were considered as non-subsistence spending.
- Out-of-pocket health expenditures (OOPE): the sum of all household members’ expenditures on health care in the month preceding the survey.
- Catastrophic health expenditures: when the sum of a household’s total out-of-pocket health expenditures equals or exceeds a threshold of the household’s capacity to pay or non-subsistence spending. We used two different thresholds: 40 and 10 %.
- Impoverishment: a non-poor household is impoverished by health payments if it falls below the poverty line after deducting health expenditures. The national poverty line set by the Ministry of Planning for rural and urban settings were used.
- Indebtedness: defined as having at least one active loan whose main purpose was to pay for the care of an illness, injury or accident.

### Statistical analysis

Data was stratified by age (younger than 60 years old, and ≥ 60 years old), and place of residence (urban/rural) and analysed using Stata version 13.1. Sampling weights were used for all individual and household-level descriptive statistics. CSES sampling weights were calibrated to reflect the accurate population composition at the time of the survey. Analysis of variance (ANOVA) was used to test for significant differences between population subgroups. When comparing monetary values over time, values for Cambodian riel (KHR) were transformed into 2014 constant values using the cumulative inflation rate of 82.0 % for the period 2004–2009 and 20.7 % for 2009–2014. During 2014, the average exchange rate of KHR to the United States dollar (US$) was KHR4,097 to US$ 1 (http://www.oanda.com/currency/average). Analysis of determinants employed logistic regression techniques, and Stata’s *svy* settings were used to account for the complex sampling methods of CSES surveys. Determinant analysis used only the subgroup of households with older people.

## Results

### Trends among older people: 2004-14

#### Demographics and health care utilisation

Table 1 provides an overview of the major characteristics of the CSES respondents in 2004, 2009 and 2014. Throughout the surveys, the number of households remained similar although the respective number of individuals decreased. The proportion of older people increased by one-third in the last decade, from 5.3 % in 2004 to 7.1 % in 2014. Older women and men had a similar mean age of 68.9 years and 68.6 years, respectively. Older people were slightly more represented in rural areas than in urban settlements: 7.3 % compared to 6.3 % (data not shown). Throughout the 10-year time period, the proportion of households with at least one older person increased from 24.2 to 28.8 %, with no difference between place of residence. Among households with older people, a quarter had two older members. The proportion of households consisting of older people living alone increased from 2.7 % in 2004 to 3.8 % in 2014.

**Table 1:** Sample characteristics

Variable	2004	2009	2014
Households	11,993	11,971	12,090
Response rates (%)	99.89	99.76	99.95
Individuals	59,852	57,105	53,968
0–59 years (%)	94.7	93.1	92.9
≥ 60 years (%)	5.3	6.9	7.1
Gender people aged ≥ 60 years			
Men	41.5	42.7	40.7
Women	58.5	57.3	59.3
Mean age in years [SD]			
0–59 years	21.4 [15.1]	23.3 [15.6]	24.8 [16.1]
≥ 60 years	68.8 [6.9]	68.9 [7.4]	68.8 [7.3]
Households with older people (in %)			
One or more older people	24.2	25.7	28.8
1 older person	18	17.6	18.8
2 older persons	6.2	5.9	7.4
Older people living alone (%)	2.7	3.3	3.8
Having a disability only (%)			
0–59 years	2.2	1.9	1.2
≥ 60 years	18.1	12.1	8.3
Having NCD only (%)			
0–59 years	NA	1.7	1.6
≥ 60 years	NA	8.4	9.7
NCD and disability (%)			
0–59 years	NA	0.4	0.3
≥ 60 years	NA	5.6	3.7
Access to drinking water (%)			
0–59 years	54.2	44.5	51.7
≥ 60 years	54.7	45.6	52.0
Have toilet (%)			
0–59 years	25.1	37.1	58.6
≥ 60 years	26.2	42.6	64.1

*NA* not assessed

The proportion of older people reporting a disability decreased considerably, from 18.1 % in 2004 to 8.3 % in 2014, although the 2014 rate is still seven times higher than among younger people. Conversely, from 2009–2014 the proportion of older people with an NCD increased from 8.4 to 10 %; six times higher than NCD rates among younger people in 2014. Older women reported more NCDs and/or disabilities than older men (23.6 % and 18.9 %, respectively). Access to drinking water remained at about 50 % throughout the concerned period, while household ownership of toilets increased drastically from 26 to 64 % over the same time.

Among older people, 34.7 % reported an illness in the month preceding interview (compared to 13 % of younger people). Older women were significantly more likely to report an illness than older men; 38.2 % and 29.5 %, respectively (*p* < 0.001; data not shown). Care seeking when sick among older people increased considerably from 86.7 % in 2004 to 97 % in 2014, with no statistical differences by age, gender or place of residence (Table 2). This increase in care seeking was especially pronounced for medical providers, which increased from 49 to 86 %. Care seeking with medical providers in 2014 was more marked among urban residents than rural ones (93 % vs. 84.3 %; *p* < 0.001; data not shown). The proportion of care sought at public facilities doubled – from 10.7 % in 2004 to 23.4 % in 2014 – but similar increases were also seen at private providers (31.9 to 64.1 %).

**Table 2:** Care seeking and associated costs for older people and comparison with younger people for selected variables 2004-14

Variable	2004	2009	2014
Incidence of illness in last 30 days			
People ≥ 60 years	38.6	33.7	34.7
People < 60 years	16.8	13.0	13.0
Seeks care when sick			
People ≥ 60 years	86.7	92.2	97.3
People < 60 years	90.8	91.3	98.2
Seeks medical care	49.0	70.1	85.9
At public health facility	10.7	18.4	23.4
At private provider	31.9	56.9	64.1
OOPE for those seeking care^a^			
People ≥ 60 years	16.8	25.6	36.6
People < 60 years	12.8	18.6	26.0
Transport for those who sought care^a^			
Rural	NA	2.8	3.9
Urban	NA	1.6	5.1

*NA* not assessed

^a^Constant 2014 Cambodian riel (KHR) converted to US$

#### Out-of-pocket health expenditures

The tendency to increasingly consult qualified providers was well aligned with the considerable increase in capacity to pay when measured in constant 2014 KHR (Table 3). There was nearly no difference in the reported capacity to pay between households with and without older people. The capacity to pay among urban households is considerably greater than the capacity of their rural counterparts. This difference decreased from 2004 to 2014, but in 2014 urban households with older people declared nearly double the capacity to pay of their rural equivalents.

**Table 3:** Trends in health financing for households with and without older people

Variable	2004	2009	2014
Capacity to pay (US$)^a^			
Households without older people	107.8	162.0	199.7
Households with older people	102.1	164.2	189.6
Rural	80.4	122.7	158.0
Urban	221.5	345.4	308.4
Out-of-pocket payments as proportion of capacity to pay (%)			
Households without older people	8.0	6.2	6.5
Households with older people	10.8	9.7	9.6
Rural	11.1	10.7	10.6
Urban	9.3	5.5	5.9
Catastrophic expenses at 40 % threshold (%)			
Households without older people	6.4	4.3	4.3
Households with older people	9.2	7.7	6.5
Rural	9.6	8.7	7.2
Urban	7.3	3.1	3.6
Catastrophic expenses at 10 % threshold (%)			
Households without older people	21.7	17.9	18.3
Households with older people	29.3	27.6	28.3
Rural	30.0	30.0	31.1
Urban	25.5	16.7	17.7
Impoverishment (%)			
Households without older people	3.2	2.3	0.9
Households with older people	4.6	2.8	1.0
Rural	4.8	3.0	0.9
Urban	3.3	2.2	1.3
Indebted for paying health care costs (%)			
Households without older people	5.0	4.0	2.5
Households with older people	4.8	3.3	2.2
Rural	5.3	3.6	2.5
Urban	2.0	2.0	1.0

^a^Constant 2014 KHR converted to US$

Expenditures on care-seeking increased substantially for both older people and younger people when measured in 2014 KHR (Table 2). In 2014, older people seeking care spent on average US$36.6 in the last month on medical care (vs. US$26.0 for younger people). Older men spent much more than older women: US$48.9 vs. US$30.0 (*p* < 0.001; data not shown). Further analysis indicated that there was no statistical difference between older men and women in the type of health provider consulted, or in the total number of consultations per month. However, the amount spent per individual consultation was significantly higher for men, US$33.1, than women, US$21.0 (*p* = 0.026). Such gender differences were also observed for transport expenses for older people who sought health care, with women spending only US$1.3 and men US$6.7 (*p* = 0.009). Transport expenses for older people in urban areas were higher than in rural areas (US$5.1 vs. US$3.9), but this was not significant.

As Table 3 indicates, out-of-pocket expenditures as a proportion of capacity to pay (OOPE/CTP) for households with older members have barely changed in the 10 years studied (from 10.8 to 9.6 %). This was especially true for rural households, which ranged from 11.1 % in 2004 to 10.6 % in 2014). Contrarily, households with older people located in urban areas experienced a considerable decrease: from 9.3 to 5.9 %.

The incidence of catastrophic expenditures for health at the 40 % threshold decreased considerably over time for households with and without older people. But in 2014, rural households with older people were still twice as likely as their urban equivalents to incur catastrophic health expenses. This difference was less pronounced at the 10 % threshold (31.1 % and 17.7 %, respectively), and the aggregated data for households with older people suggests no significant change over time at this threshold. Indebtedness due to health care expenses decreased significantly for households with older people. The proportion of households indebted because of paying healthcare costs halved over the study period, irrespective of the presence of older people and place of residence. Incidence of indebtedness decreased more in rural households than urban ones. However, by 2014 rural households with older people were still two and a half times more likely to be indebted than equivalent urban households, which was similar to the situation 10 years earlier.

### Determinants of catastrophic health expenditures for households with older people

Table 4 provides an overview of the multivariate regression analysis concerning determinants of catastrophic health expenses for older people at the 10 % and 40 % thresholds.

**Table 4:** Odds ratios from multivariate logistic regressions identifying associations between selected household characteristics and catastrophic health expenditure

Variables	40 % Threshold	10 % Threshold
	OR (T-statistics)	
Household size	0.94 (−1.29)	0.94 (−2.01)*
Number of children under five years old	1.29 (1.37)	1.48 (3.82)**
Number of older members	1.30 (1.43)	1.28 (2.08)*
Age head of household	1.01 (1.34)	1.01 (1.60)
Education of head of household		
Primary education or more	1.00 (.)	1.00 (.)
Less than primary education	1.00 (−0.02)	1.33 (2.28)*
Gender of head of household		
Female	1.00 (.)	1.00 (.)
Male	0.71 (−1.74)	0.84 (−1.32)
Place of residence		
Urban	1.00 (.)	1.00 (.)
Rural	2.81 (3.09)**	2.99 (5.83)**
Household with priority access card		
No priority access card	1.00 (.)	1.00 (.)
Priority access card	1.43 (1.47)	1.08 (0.44)
Household member with a mild illness		
None	1.00 (.)	1.00 (.)
At least one	1.95 (2.83)**	6.48 (15.06)**
Household member with severe illness		
None	1.00 (.)	1.00 (.)
At least one	2.45 (3.84)**	5.16 (7.77)**
Household member with a disability		
None	1.00 (.)	1.00 (.)
At least one	1.02 (0.08)	1.24 (1.60)
Household member with a chronic disease		
None	1.00 (.)	1.00 (.)
At least one	5.04 (7.16)**	13.85 (18.78)**
Access to improved water source		
Unimproved	1.00 (.)	1.00 (.)
Improved	0.88 (−0.66)	0.95 (−0.50)
Household having a toilet		
No access	1.00 (.)	1.00 (.)
Access	0.54 (−3.16)**	0.97 (−0.25)
Remittances from relatives		
No remittances	1.00 (.)	1.00 (.)
Receives remittances	1.15 (0.75)	1.15 (1.29)
Pension, social welfare benefits		
No pension or social welfare benefits	1.00 (.)	1.00 (.)
Receives pension or social welfare benefits	0.63 (−1.05)	0.73 (−1.16)
Transfers from NGO or other institutions		
No transfers	1.00 (.)	1.00 (.)
Receives transfers	0.57 (−0.86)	0.73 (−0.87)

*OR* odds ratio

*Significant at 5 % (*p* < 0.05)

**Significant at 1 % (*p* < 0.01)

Catastrophic health expenditures correlated with the presence of more children in the household, although only for the 10 % threshold. Residing in rural areas significantly increased the likelihood of catastrophic expenses, in comparison with urban households. One of the most important factors for catastrophic expenses at both thresholds was the presence of a household member with an NCD. However, even the presence of a member with a mild illness increased the likelihood of catastrophic health expenditures in households with older people. This was also the case when a household included a member with a severe illness.

The presence of a toilet greatly protected against catastrophic health expenses at the 40 % threshold. Having a disabled person in the household did not affect the likelihood for such expenses.

Contrary to its intent, having a priority access card to receive free health care in public health facilities at the point of delivery – a benefit possessed by 8.9 % of households with older people – did not have a statistically noticeable effect on catastrophic health expenditures. Other social assistance interventions such as welfare benefits (5.2 % of households with older people), or cash transfers by third parties (2.4 %) did decrease the likelihood for catastrophic health expenses, although this effect was not statistically significant. Remittances from household members, received by 56.5 % of households, also had no significant effect on catastrophic health expenses.

## Discussion

As expected, the share of older people in the Cambodian population has increased over time. Throughout the documented time period, about a quarter of households had at least one older member. A small proportion of households consisted of older people living alone. Because the resulting sample size was too small for meaningful statistical analysis, the conditions among these households were not further investigated. Older people living alone or without relatives, however, tend to have poorer physical and mental health, and thus a lower quality of life, than those living with relatives [4].

The prevalence of self-reported disabilities among older people has decreased drastically over time although one in 10 older people reported having an NCD. The CSES questionnaire did not elicit the nature of reported chronic conditions, but other studies have indicated a high prevalence of diabetes and hypertension among the older Cambodian population. A survey conducted in 2004 found diabetes rates of 5 % in a rural setting and 11 % in an urban environment, while respective figures for hypertension were 12 and 25 % [15]. Moreover, two-thirds of diabetics and more than half of those with hypertension were unaware of their conditions, suggesting that the CSES prevalence rates may be an underestimation.

Despite the 19–24 % of older people in the sample reporting an NCD or disability, and the fact that both conditions are associated with a tendency for catastrophic health expenditures [10], the trend in financial access to care for older people during the studied time period appeared very positive. As such, care-seeking by older people with an illness was nearly universal, care was increasingly sought from medical providers, and use of public health facilities became more prominent.

However, the amount spent on medical care more than doubled over the decade. Such expenditures were apparently well absorbed, as the capacity to pay increased considerably for households with older people. Nevertheless, OOPE/CTP remained nearly the same throughout the concerned period, although the incidence of catastrophic expenditures for health care decreased, as did impoverishment due to healthcare expenses and health-related indebtedness.

As thresholds for the definition of catastrophic health expenses vary among scholars [16–18], we also applied the 10 % threshold as Brinda and colleagues found that, in India, older people who spent more than 10 % of their income on health were more likely to face food scarcity and have suicidal tendencies [18]. Applying this threshold provides a less positive picture, especially for rural households with older people, as the incidence of catastrophic health expenses at this threshold remained the same throughout the observed period. Similarly, while OOPE/CTP decreased for urban households with older people during that time, this was not the case for rural households.

Rural households with older people also had double the risk of incurring catastrophic health expenses at the 40 % threshold than their urban equivalents and were two and a half times more likely to be indebted because of health care-related expenses. Multivariate regression analysis confirmed the significantly increased likelihood for catastrophic expenses among rural households with older people. Rural residence was also an important factor for catastrophic health expenditures among older people in China and India [19].

Despite improvements in financial protection over time, households with older people remained more susceptible to catastrophic health expenses than other households. This may be because older people experienced a much higher incidence of illness than younger people and spent more than younger people for health in the month preceding interview. Such higher costs can be ascribed to the complex treatment requirements of older people, as they tend to suffer from multiple conditions [4, 12].

Despite being more likely to report an illness or have a disability or NCD, older women spent significantly less on health care than men, which is similar to observations from India [18]. Van der Meulen Rodgers [20] analysed the 2005 Cambodia Demographic and Health Survey data and found less care-seeking among older women than men. He ascribed this to women’s lower educational attainments and a greater tendency to live with multiple children, with whom they may have to share resources. Multivariate regression analysis of the 2014 CSES data indicates that the probability of incurring catastrophic health expenditures at the 10 % threshold increased significantly with the number of cohabiting children. As such, although they sought care to the same extent as older men, older women may have selected cheaper treatment options and sought treatment closer to home to save on transport costs, as indicated by their much lower transport expenses.

The financial vulnerability of households with older people is signified by the fact that having a member with a mild illness in the month preceding interview significantly increased the changes for catastrophic health expenses. These odds ratios were higher at the 40 % threshold for severe illness, and highest when a household member reported an NCD. As mentioned, NCDs are major determinants for health spending in other Asian countries as well [3, 4, 8, 10, 18]. While NCDs constituted a major determinant for catastrophic health expenses, having a disability did not, unlike findings from Thailand and India [10, 18]. This may be due to the fact that people with a disability accessed health services to a lesser extent than people without, as reported elsewhere [21, 22]. Alternatively or additionally, since sight-related disabilities were excluded due to inconsistencies in the data, the ability to identify a statistical relationship between reporting a disability and catastrophic health expenses may have been reduced.

There was no statistical effect of social assistance support, such as a pension, social welfare, and support from nongovernmental organisations (NGOs), on catastrophic health expenses among households with older people, suggesting inadequacy in these interventions. Nearly one in 10 households with older people had a priority access card which entitled them to free health services at public facilities at the point of delivery under the health equity fund scheme, a third party arrangement that reimburses public health providers for services rendered to the poor. However, older people benefiting from this scheme did not seem to enjoy the financial protection they were supposed to receive. This may be due to costs incurred for treating NCDs, as a recent assessment of the financial protection offered by social health protection schemes for people with diabetes and hypertension indicated the inadequacies of such schemes, whereby a large proportion of recipients were indebted as a result of care-seeking and only half were receiving medical treatments for NCDs [23]. While such social health protection schemes typically contract public health facilities to provide services to their beneficiaries, another study indicated that the public health sector in rural Cambodia is inadequately equipped to successfully manage NCDs [24].

Receiving remittances also did not buffer households against healthcare costs; possibly because the remittance amounts were too small, or devoted to other necessities by the recipients. A survey from 2008 found that workers in the garment industry – which absorbs a considerable proportion of the rural youth that migrate to towns–sent US$ 15 or 21 % of their monthly income to their families [25]. While these remittances may not have been sufficient to reduce the likelihood of catastrophic health expenditures among recipients, the support provided to these families appears to be considerable. This is in line with Asian cultural values, which consider support for dependent parents as a norm [6, 26]. These practices do not seem to have suffered from urbanisation and migration, despite an annual emigration from rural villages of 4.8 %, half of which is permanent [27].

While access to clean drinking water had no statistically significant protective effects against incurring costs for health care, having a toilet greatly reduced the likelihood of catastrophic health expenses, which is similar to findings from India [18]. This effect was most likely due to a resulting lower incidence of infectious diseases. It is therefore positive to note that the share of households with a toilet rose from 26 % to 64 % over the ten-year period studies, although a third of households were still without toilet access in 2014. Investments in sanitation will not only help protect older people against the costs of illness, but also advance the health of relatives, especially children [28].

### Limitations

Data used for this overview was derived from routine surveys that were not specifically designed for collecting information related to health financing. The recall period applied in the three surveys related only to the month prior to the interview. As such, conditions of a serious nature which have a low frequency but require comprehensive – and thus more expensive – treatments may have been missed, leading to an underestimation in the incidence of catastrophic health expenditures and impoverishing healthcare costs. As sight-related difficulties were not included in the disability analysis, their financial impact on respective patients is unclear. Not considering sight-related disabilities may be a reason why this analysis did not find a statistical correlation between disabilities and catastrophic health expenditures. The depth of poverty was also not estimated, which is the degree by which OOP for health pushes already poor households further below the poverty line [29]. However, data were collected using similar research instruments during the concerned time periods, thereby maintaining consistency throughout, and enabling the observation of trends.

## Conclusion

In line with other countries in the region, the older population in Cambodia is rapidly increasing. A considerable proportion of such people suffer a disability or NCD. The proportion of older people seeking care when ill has increased considerable from 2004 to 2014, and such care was increasingly sought from medical providers. A preference was noted for private practitioners, although public health facilities were also increasingly consulted. While the financial consequences of health care have lessened over time, households with older people, especially rural ones, remained disadvantaged as OOPE/CTP stayed the same throughout the period studied, despite increased incomes. Aside from rural residence, major determinants of catastrophic health expenditures were the presence of a household member with any illness in the month preceding the interview, especially chronic illnesses. Available social assistance interventions as well as remittances by relatives did not offer any statistically significant protection against catastrophic health expenditures. However, having a toilet was conducive to lower the chance for catastrophic expenses.

These findings imply that in order to make health care more equitable for older people, efforts should be directed to rural areas. Interventions should include improving management of NCDs at the primary care level in public health facilities, together with a reconfiguration of social health protection schemes to focus particular attention on the inclusion of older people.

## Abbreviations

CTP, capacity to pay; HH, household; KHR, Cambodian riel; LMIC, low- and middle-income country; NCD, non-communicable disease; NGO, nongovernmental organisation; OOP, out-of-pocket (health expense); US$, United States dollar
